# Altered Food Behavior and Cancer: A Systematic Review of the Literature

**DOI:** 10.3390/ijerph191610299

**Published:** 2022-08-18

**Authors:** Daniele Nucci, Omar Enzo Santangelo, Sandro Provenzano, Mariateresa Nardi, Alberto Firenze, Vincenza Gianfredi

**Affiliations:** 1 Nutritional Support Unit, Veneto Institute of Oncology IOV-IRCCS, Via Gattamelata, 64, 35128 Padua, Italy; 2 Regional Health Care and Social Agency of Lodi, ASST Lodi, Piazza Ospitale, 10, 26900 Lodi, Italy; 3 Local Health Unit of Trapani, ASP Trapani, 91100 Trapani, Italy; 4 Department of Health Promotion, Mother and Child Care, Internal Medicine and Medical Specialties (ProMISE) “G. D’Alessandro”, University of Palermo, Via del Vespro, 133, 90127 Palermo, Italy; 5 Department of Biomedical Sciences for Health, University of Milan, Via Pascal, 36, 20133 Milan, Italy; 6 CAPHRI Care and Public Health Research Institute, Maastricht University, 6200 MD Maastricht, The Netherlands

**Keywords:** cancer, food addiction, orthorexia nervosa, cancer survivors, food craving, compulsive eating, food behavior, systematic review

## Abstract

There is evidence of an association between cancer and certain types of altered eating behaviors, including orthorexia, food cravings, and food addiction. Given the growing interest in the topic throughout the scientific community we conducted a systematic review to summarize current evidence on the development of altered food behavior, including food addiction and cancer. The Cochrane Collaboration and the Meta-analysis Of Observational Studies in Epidemiology guidelines were followed to perform this systematic review. The Preferred Reporting Items for Systematic Reviews and Meta-Analyses (PRISMA) statement was used to report the process and results. The structured literature search was conducted on 19 April 2022, on PubMed/Medline and Scopus, combining free-text terms and medical subject headings. A total of seven articles were included once the selection process was completed. Food craving has been associated with different types of cancer in adults and young patients, as well as with orthorexia; conversely, compulsive eating has only been explored in patients with prolactinoma treated with dopamine agonists. This systematic review explored a new area of research that warrants further investigation. More research is required to better understand the relationship between cancer and food behavior.

## 1. Introduction

According to the World Health Organization (WHO), cancer caused nearly one in six deaths in 2020, representing the leading cause of death worldwide [1,2]. Nevertheless, important advancements in prevention, diagnosis, and treatment have substantially increased survival rates [3]. The WHO has estimated that one in three new cases of cancer may be prevented by adopting a healthy lifestyle [4]. Lifestyle factors, such as smoking, alcohol consumption, obesity, sedentary behavior, and poor diet are all regarded as cancer risk factors and thus primary and secondary prevention objectives [5,6]. Diet is one of the factors that has garnered more attention in the last decade. In fact, clinicians, the public, and patients are focusing more on diet in both prevention and after cancer diagnosis [7,8,9]. Recent evidence suggests that cancer is linked to eating habits and obesity/overweight in the long term [10,11]. In light of the importance of diet and the growing number of cancer survivors, it is essential to investigate cancer survivors’ eating behaviors and, ultimately, how these can affect survival rates and quality of life. 

Evidence suggests there is a link between cancer and certain types of altered eating behaviors, such as orthorexia, food cravings, and food addiction [12,13,14]. Orthorexia is an obsession with healthy and proper eating [15], food cravings are an intense desire for a certain food [16], but food addiction is a rather more complex concept. Food addiction is defined as a behavioral addiction that is characterized by the compulsive consumption of palatable foods (e.g., foods containing high concentrations of refined sweeteners, flour, fats, salt, and/or caffeine) that markedly activate the reward system in humans and other animals despite adverse consequences, without the need to focus purely on psychoactive substances [17]. Food addiction was not recognized in the fifth edition of the Diagnostic and Statistical Manual (DSM-V); however, similarities between certain eating disorders and substance-use disorders have been acknowledged (such as reduced control over intake, increased impulsivity, and altered reward-sensitivity) [18]. Indeed, food addiction has been implicated as a putative causal factor in chronic overeating, craving, binge eating, and obesity; therefore, the model of addiction on processed food is a biologically plausible explanation for this overconsumption as a loss of control over the intake of a particular substance or behavior, without the need to focus purely on psychoactive substances [19,20]. 

In view of the considerable increase in cancer survival rates, the importance of diet in cancer, and the growing interest in the association between cancer and diet, we conducted a systematic review to summarize current evidence on the development of altered food behavior (including food addiction) and cancer. 

## 2. Materials and Methods

The Cochrane Collaboration [21] and the Meta-analysis Of Observational Studies in Epidemiology (MOOSE) guidelines [22] were followed to conduct this systematic review. The Preferred Reporting Items for Systematic Reviews and Meta-Analyses (PRISMA) statement, published in 2020, was used to report the process and results [23]. A standardized protocol identifying the research question, the search strategy, and inclusion and exclusion criteria was developed and shared within the research team and fully approved before starting the review.

### 2.1. Data Sources and Search Strategy

The structured literature search was conducted on PubMed/Medline and Scopus on 19 April 2022, combining free-text terms and medical subject headings (MeSH). The Boolean operators AND and OR were used to combine keywords. No time filter was applied. A blind search strategy, which was specific for each database, was conducted by two authors (VG and DN). The full search strategy is available in Supplementary Table S1. Additional relevant articles were identified through manual inspection of reference lists. Professionals involved in the field were also consulted to facilitate the collection of any other potentially relevant material.

### 2.2. Eligibility Criteria

In order to be considered eligible, articles had to meet the following inclusion criteria: (i) an English-language article, (ii) reporting original data, (iii) a focus on food addiction, and (iv) a focus on cancer/neoplasm.

Exclusion criteria included: (i) no original data (review, opinion, book chapter, commentary, or letter), (ii) article published in a language other than English, (iii) no full-text available, and (iv) assessing outcomes unrelated to food addiction among cancer patients.

Table 1 depicts a detailed description of inclusion and exclusion criteria based on the Population, Exposure, Outcomes, and Study Design (PEOS), adjusted for observational studies extended with time and language filters, as recommended by the Cochrane Collaboration.

### 2.3. Selection Process and Data Extraction

Similar to previous research [24], article screening for this systematic review was performed manually and with the EndNote^®^9.0 (Clarivate^TM^, Philadelphia, PA, USA) software. The screening process was conducted in two steps. The first selection was based only on title and abstract. Article screening was individually conducted by two authors (VG and DN). Full text was only downloaded for studies that met all the criteria. In both phases, any disagreement between reviewers was debated until consensus was reached. As in previous studies [25,26], double-blind data extraction was performed (VG and DN) using a predefined spreadsheet processed in Microsoft Excel^®^ for Windows (Microsoft Corporation, Redmond, Washington, DC, USA). The spreadsheet was pre-piloted on two randomly-selected articles to standardize data extraction [27,28,29]. Extracted data included: First Author, Year, Country of Study, Study Period, Study Design, Main Characteristics of the Sample, Sample Size, Attrition, Type of Altered Food Behavior, Diagnostic Tool for Altered Food Behavior, Validated Diagnostic Tool (for altered food behavior), Number of Subjects with Altered Food Behavior, Main Results, Funds, and Conflict of Interest. Any disagreement regarding data extraction was resolved by discussion between the two authors; if the disagreement persisted, a third author was consulted (OES). A fourth author (MN) conducted random checks.

### 2.4. Quality Assessment

The quality of included studies was assessed based on the study design. The Newcastle-Ottawa Quality Assessment Scale (NOS) [30], a validated tool developed to check the methodological quality of observational studies, was used to assess case–control and cohort studies. An adapted version of the NOS was used to assess cross-sectional studies [31]. In accordance with previous research [6,29], studies were classified as being of high, moderate, or low quality when their NOS score was ≥7, 4–6, and ≤3, respectively. The “JBI Checklist for Case Reports” [32] was used for case reports. Finally, the Risk of Bias-2 (RoB-2) of the Cochrane Collaboration tool for randomized trials was utilized to assess the quality of trials [33]. The quality assessment of randomized trials only allows for a qualitative rating without quantitative results, ranging between high risk of bias, some concern, and low risk of bias. Similarly, the rating for pre–post interventions can be good (if score ≥75%), fair (score between 75% and 25%), and poor (if score ≤25%). The quality assessment of included publications was carried out individually by two authors (OES and SP).

### 2.5. Analysis and Presentation of Results

As previously described [25,34], extracted data were used to report the key findings in tabular and synthetic form. The data collected, retrieved, and evaluated in this review were used to evaluate the possible association between food addiction and all types of cancer.

## 3. Results

### 3.1. Literature Search

**Figure 1:**
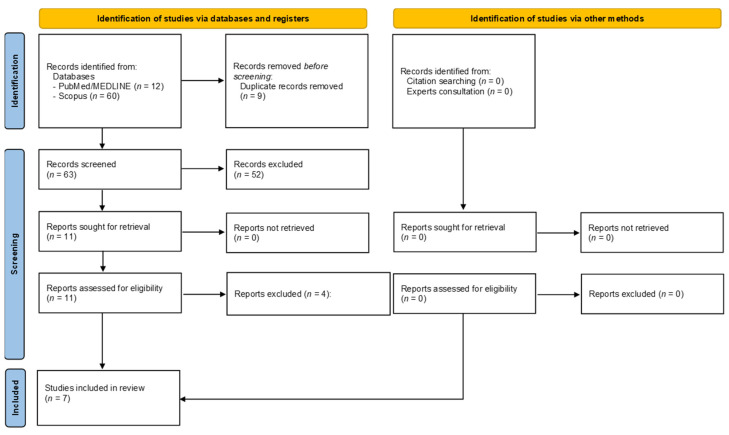
Flow diagram depicting the selection process.

We identified a total of 72 articles: 12 from PubMed/Medline and 60 from Scopus. A total of nine articles were immediately removed due to duplication and two additional studies were excluded because they were not written in English. Another 50 studies were removed once the selection criteria were applied. After title and abstract screening, a total of 11 articles were considered eligible and read through. However, after the full-text assessment, four additional records were removed with justifications. The reasons for exclusions are reported in Supplementary Table S2 [35,36,37,38]. A total of seven articles were included in this systematic review once the full selection process was completed [12,13,14,39,40,41,42]. Figure 1 illustrates the selection process. 

### 3.2. Characteristics of Included Studies

Table 2 describes the main characteristics of the seven included studies [12,13,14,39,40,41,42]. In terms of study design, the majority (*n* = 3) of the studies were cross-sectional, with one multicenter study involving 11 tertiary referral centers. One case–control study, one prospective cohort study (with a 12-month follow-up), one randomized clinical trial with a parallel arm, and one case report rounded out the remaining studies. Approximately half of the studies (*n* = 3) were conducted in the United States of America (USA), followed by Turkey (*n* = 2), and Canada and Slovakia for the remainder. The two most recent studies were published in 2020 [12,39], while the first study on this topic was published in 2011 [14]. The largest study included 238 female breast cancer cases and 164 healthy controls, for a total of 402 subjects [12], followed by 308 prolactinoma patients [41]. All the remaining studies had between 20 and 40 subjects, with the exception of the case report which described a patient’s medical history [39].

### 3.3. Characteristics of Participants

According to the inclusion criteria, all the participants had been diagnosed with cancer: two studies were conducted on women with breast cancer [12,42], two studies included subjects with pituitary adenomas (prolactinoma) [14,41], two studies dealt with hematological neoplasia in young survivors of pediatric acute lymphoblastic leukemia (ALL) and lymphoma [13], and one included elite athletes with acute lymphoblastic leukemia [39]. The remaining studies focused on women with ovarian cancer [40]. All the studies involved adults, with the oldest population (60.2 years; range 31–79 years) recruited by Cohen et al., with the exception of one study involving a pediatric population [13] with a mean age of 11.7 years (range 4.7–24.9 years). The entire study reported no attrition (all subjects completed all the assessments and were included in the formal analysis), but the RCT reported an attrition of 28 participants [40]. The trial was registered at ClinicalTrials.gov as NCT03171506.

### 3.4. Altered Food Behavior Examined

Food craving was the most frequently assessed food addiction (*n* = 3) [13,40,42], followed in equal measure by orthorexia nervosa (*n* = 2) [12,39], and compulsive eating (*n* = 2) [14,41]. Two of the three studies assessed food craving using the validated Food Cravings Inventory tool, while the third employed a semi-structured qualitative interview and validation was not reported in the article. In both investigations, orthorexia nervosa was assessed using the Orthorexia Nervosa Scale (ORTO-15), which is a validated tool. Lastly, compulsive eating was assessed by means of different tools. One study used the Minnesota Impulse Disorder Interview (MIDI) [14], while the other employed the Questionnaire for Impulsive-Compulsive Disorders in Parkinson’s disease (QUIP) [41]. Both are validated tools, with the MIDI having been validated using the Diagnostic and Statistical Manual of Mental Disorders, Fourth Edition. 

### 3.5. Altered Food Behavior and Cancer

Food cravings have been found to be associated with breast cancer, pediatric ALL and lymphoma, and ovarian or endometrial cancer. 

The results obtained indicate that food cravings in children with cancer are detected more frequently when patients are diagnosed at an older age. Moreover, patients had higher cravings overall and for specific food subscales such as fast foods, sweets, carbohydrates (starchy food), and fats [13]. In a cohort of female breast cancer patients, similar food subscales were found to be associated with food cravings during treatment [42]. In both studies, food cravings were responsible for weight gain and obesity. However, the only RCT identified in the literature found that after 12 weeks of the ketogenic diet (KD), women with ovarian or endometrial cancer reported less frequent cravings for starchy foods and fast food than the group on the American Cancer Society (ACS) diet. The KD group also reported fewer cravings for starchy foods, sweets, fast food, and overall cravings at 12 weeks compared to baseline. These differences were not found when the ACS group was taken into consideration.

Compulsive eating was only identified in patients with prolactinoma treated with dopamine agonists. Both studies [14,41] found an association between compulsive eating and a high level of prolactin, specifically a high nadir prolactin level. In addition, compulsive eating was associated with other unhealthy behaviors such as smoking, alcohol consumption, and a history of gambling. In both studies, compulsive eating was associated with body weight gain.

Lastly, orthorexia nervosa was assessed in patients with breast cancer and acute lymphoblastic leukemia. Results show that the risk of orthorexia is higher in patients who are highly educated, eat organic foods, have other chronic diseases (other than cancer), and have received care support [12]. Orthorexia nervosa may result in weight loss malnutrition and interpersonal impairment [39].

### 3.6. Quality Assessment

According to the defined cut-points, all of the articles were deemed to be of moderate to high quality. Two studies scored nine (high) [12,41], one study scored 10 (high) [13], and the remaining two totaled four points (moderate) [14,42]. The main concerns centered on the selection of controls (Item 3), which was not fully described in three studies [14,41,42], the comparability of any additional factors [14,42], and the quality of the statistical test (Item 7), which was not adequately described in two studies [14,42]. We included Bobonis Babilonia et al.’s [39] case report since it satisfied all the “JBI checklist for case report” criteria. For the randomized clinical trial by Cohen et al. [13], the “Risk of Bias-2 (RoB-2)” tool showed a low risk of bias. All details are reported in Supplementary Table S3.

Three studies did not specify whether any financial support had been granted [12,14,39], whereas the other three reported receiving financial support. However, none of the included studies disclosed any conflict of interest (Table 2). 

## 4. Discussion

The data analyzed in this report are the result of a systematic review conducted on the main scientific databases: PubMed/MEDLINE and Scopus. The search identified 62 articles, excluding duplicates, with a total of seven articles included in the analysis at the end of the screening process. An evaluation of the selected articles revealed that the topic is relatively new, with the first study published in 2011. However, there is growing interest in the topic throughout the scientific community, as all the other articles on the subject were published within the past five years (approximately). To date, three main areas of altered food behaviors have been explored in association with cancer: food craving, compulsive eating, and orthorexia. Food craving has been associated with different types of cancer in adults and young patients, as well as with orthorexia; conversely, compulsive eating has only been explored in patients with prolactinoma treated with dopamine agonists. It is commonly assumed that cravings are an “expression of an energy or specific nutrient requirement” [16], whereas compulsive eating is defined as “repetitive bouts, without homeostatic function, with adverse consequences, and as a way to relieve stress” [43]. These differences may be attributed to a potentially diverse biological mechanism. Compulsive eating is well documented as a complication of dopamine agonist treatment, previously described in Parkinson’s disease [44], but also in schizophrenia [45]. In these patients, the dopamine agonists cause an excessive and aberrant activation of the mesolimbic dopaminergic system which is, in turn, responsible for the compulsive eating behavior [46]. 

Food cravings and compulsive eating can vary in intensity and frequency among individuals. Several studies found that people who have the most frequent and intense food cravings have a tendency to lose control during eating and have poor weight management, making them more prone to being overweight or obese [47,48,49]. Obesity is a leading risk factor for at least 13 different cancers (mouth, pharynx and larynx, esophagus, breast, stomach, pancreas, liver, gallbladder, kidney, colorectum, endometrium, ovary, and prostate) and increases cancer-related mortality [50]. Moreover, obesity may impair the efficacy of anti-cancer treatment [51], increase the risk of cancer recurrence, obesity-related comorbidities (such as cardiovascular diseases, insulin resistance, glucose intolerance, diabetes mellitus, and hypertension), and overall mortality. It may also lower cancer survivors’ quality of life [50]. Maintaining body weight within a normal range or implementing actions to support people in losing weight, as recommended by the World Cancer Research Fund (WCRF) and the America Institute for Cancer Research (AICR), is a compelling evidence-based strategy for lowering cancer risk [50]. Given that food craving may be responsible for the ineffectiveness of weight loss interventions, early diagnosis and reduced food cravings may lead to successful weight loss and contribute to tackling obesity [52]. 

By definition, orthorexia is not an eating disorder, since healthy nutrition habits act as protective behaviors against several acute and chronic diseases. However, becoming overly involved in food selection, preparation, and consumption to the point where daily activities are (severely) disrupted can be considered a pathological change in behavior and personality [53]. In this case, orthorexia becomes an obsession in “extreme dietary purity,” associated with anxiety to improve health and prevent diseases by means of a proper diet [54]. This said, a proper diet is not always a healthy diet. In fact, in most cases, subjects may decide to completely avoid certain foods while severely limiting others [53]. This food restriction is often associated with a higher risk of weight loss, nutrient deficit, and malnutrition, even though there is no intention to lose weight. This condition is very risky for cancer patients for whom a healthy diet and an appropriate nutritional status are fundamental during both treatment and the follow-up period [55], and more so when one considers that approximately 20% of cancer patients die of malnutrition [56,57]. In this perspective, appropriate dietary counseling and nutritional evaluation are extremely important to avoid harmful pressure to comply with healthy dietary recommendations. In reality, the media has raised more awareness on diet and health, also influencing the food choices of the general public and of those with chronic diseases such as cancers [7]. Cancer patients may pay more attention to their lifestyle (including diet) because they would like to ameliorate, not to deteriorate, their health [8,9,58]. It is also important to note that the impressive spread of the internet and the abundance of (pseudo) medical information available on the web may expose cancer patients to misinformation and misinterpretation [59], thereby increasing the risk of inappropriate eating behavior, which may be a precursor to eating disorders.

In light of the above, it is important to assist cancer patients not only from a strictly oncological standpoint, but also for them to be appropriately screened for any type of food alterations, nutritional status, and to educate them on the importance of their diet. 

### 4.1. Implications for Public Health Policies and Practice

Regarding public health and preventive strategies, this study suggests that altered food behavior is quite common among cancer patients and survivors, even if it is not currently evaluated as part of a routine health assessment. Cancer patients may exhibit different types of altered food behaviors, and these may be due to hormonal and pharmacological effects or be influenced by socio-cultural and environmental aspects. In fact, the high awareness raised by the media on diet and health, the abundance of (dis)information on the web, and the support of family members and healthcare workers are all important factors that can influence food behavior. Previous research found an association between media events and an increase in web searches for information about diets and recipes, suggesting that the media may play a role in food-related behavior and interest. Moreover, social support is associated with better disease management, especially for chronic diseases [60,61,62], such as cancer. However, one of the included studies found a statistically significant association between orthorexia and receiving care support in breast cancer patients [12]. An explanation for these apparently contrasting results may lie in the fact that the authors adopted the ORTO-15 questionnaire, which does not distinguish between healthy eating and pathologically healthy eating. 

In light of this, we can speculate that primary prevention interventions, such as adopting screening tools in clinical practice, educating clinicians and caregivers in supporting cancer patients on their food choices, such as sharing the same meals, might be useful to improve dietary habits, control food behavior, and lower the short- and long-term risk of eating disorders. Furthermore, these primary prevention interventions may also have some secondary prevention effects. Cancer patients and survivors who follow a healthy diet stand to benefit from an improved treatment response and quality of life, and a potential lower risk of cancer relapse. 

As to public policies, our data contributes to the investigation of the association between cancer and food behavior. Cancer is a leading cause of global disease and death, with 22 million cases projected over the next 20 years [4]. For this reason, solid evidence needs to be collected and then implemented in public health. 

### 4.2. Strengths and Limitations

Some strengths and limitations should be examined. First, this is a systematic review limited to only two databases, as required by the PRISMA guidelines for systematic reviews. Second, we limited our search to articles published in English, given that English is the most frequently used language in science. However, no eligible articles were removed because of language restrictions. We are, therefore, confident that there was no selection bias attributable to these inclusion criteria. Third, there is a paucity of evidence on this topic and the level of heterogeneity is high in terms of study design, where both observational and experimental trials were found. Nevertheless, the systematic nature of this review enabled us to gather all available evidence, which resulted in the inclusion of only seven articles in total. This underlines the novelty of the topic and the need for new research. Indeed, more research on the relationship between cancer and altered food behavior could be useful in both clinical and public health settings, especially given the high cancer burden worldwide.

## 5. Conclusions

To conclude, the current systematic review explored a new area of research that warrants further investigation. Given the nature and very limited number of available studies, extreme caution is required when drawing conclusions. More research is required to better understand the relationship between cancer and food behavior. In fact, our findings highlight that there is still a significant knowledge gap. A better understanding of food behavior among cancer patients and survivors is of paramount importance to provide appropriate and multi-professional healthcare support. We, therefore, strongly recommend food behavior screening for cancer patients, both at the initial visit and during the follow-up period, in order to promptly detect any potential alteration in food behavior.

## Figures and Tables

**Table 1:** Detailed description of inclusion and exclusion criteria based on a Population, Exposure, Outcomes, and Study Design (PEOS).

Inclusion Criteria	
P = population	Patients (both pediatrics and adults) with a clinical diagnosis of altered food behavior
E = exposure	Altered food behavior directly due to cancer
O = outcome	Association between cancer and altered food behavior
S = study design	Original epidemiological studies (case-control, cross-sectional, or cohort studies) and clinical trials
**Exclusion Criteria**	
P = population	Subjects with a clinical diagnosis of altered food behavior due to other medical conditions
E = exposure	Altered food behavior indirectly due to cancer, e.g., food alteration
O = outcome	Association between cancer and other factors
S = study design	Article review, systematic reviews, meta-analysis, expert opinions, commentary, and articles with no quantitative information or details

**Table 2:** Main characteristics of included studies, reported in alphabetical order (surname of the first author).

Author, Year [Ref]	County	Study Period	Study Design	Main Characteristics of the Sample	Sample Size (% of F); Age as Mean ± SD	Attrition	Altered Food Behavior	Tool Used to Diagnose Altered Food Behavior	Validated Diagnostic Tool	*n* (or %) of Subjects with Food Addiction	Main Results	Funds	CoI
Aslan et al., 2020 [12]	Turkey	May 2018 and March 2019	Ca–Co	Inpatient adult women diagnosed with breast cancer vs. cancer-free adult women enrolled in a primary health center	Ca: 238; 49.0 ± 9.8 y Co: 164; 47.6 ± 12.5 y	0	Orthorexia nervosa	ORTO-15	yes	Ca: 23.3% Co: 6.7%	Risk of orthorexia was higher for those with a higher educational level (university degree), receiving care support, presence of a chronic disease other than cancer, and a diet that includes eating organic foods.	ns	no
Bobonis Babilonia et al., 2020 [39]	USA	ns	Case report	Elite athletes with acute lymphoblastic leukemia	30 y (sex not specified)	na	Orthorexia nervosa	ORTO-15	yes	na	The desire to eat “healthy” (with juicing and a daily meal) reported as “normal” led to weight loss, malnutrition, interpersonal and sports-related impairments.	ns	no
Cohen et al., 2018 [40]	USA	October 2015 and April 2017	RCT assessing the role of KD vs. ACS on physical and mental health, including food cravings	Women diagnosed with ovarian or endometrial cancer	45; 60.2 (31–79 y)	28	Food craving	FCI	yes	na (mean values were reported)	At 12 weeks after the intervention, the KD group reported less frequent cravings for starchy foods and fast food than the ACS group. The KD group reported less frequent cravings for starchy foods, sweets, fast food, and overall cravings at 12 weeks when compared to baseline. No within-group differences for the ACS group.	yes	no
Dogansen et al., 2019 [41]	Turkey (11 tertiary referral centers)	ns	Multicenter CS	Adult patients with prolactinoma receiving DA for at least three months	308 (70.1% F); 36 ± 12 y	0	Compulsive eating	QUIP	yes	9 (2.9% all F)	A higher QUIP score was associated with smoking, alcohol consumption, a gambling history, and a higher nadir prolactin level.	no	no
Martinkova et al., 2011 [14]	Slovakia	January–December, 2009	CS	Inpatient adults with pituitary adenomas who were taking DA	20 (50% F); 41.3 ± 11.0 y	0	Compulsive eating	MIDI	yes, based on DSM-IV criteria	1 (5% M)	Compulsive eating, especially at night, which resulted in a weight gain of 20 kg in two years. High prolactin level (16,193 ng/mL)	ns	ns
Shams-White et al., 2016 [13]	USA	ns	Co (12-month FU)	Young survivors of pediatric acute lymphoblastic leukemia (ALL) and lymphoma	22 (32% F); 11.7 (4.7–24.9) y		Food craving	FCI	yes	na (mean values were reported)	Patients diagnosed at an older age had more frequent cravings overall and for each of the subscales: fast food, sweets, carbohydrates, and fats.	yes	no
Vance, Campbell et al., 2017 [42]	Canada	ns	CS	Women with stage I–IIIA breast cancer within 12 months of completing chemotherapy treatment	28 (100%); 48.9 ± 8.5	0	Food craving	Semi-structured qualitative interview	ns	6 (50%) women who gained weight during treatment	Almost half the women who gained weight during treatments recalled that they ate more frequently and preferred starchy, carbohydrate-rich foods and others (ice cream, chocolate milk, citrus fruits, and cheese).	yes	no

ACS: American Cancer Society diet; Ca-Co: case–control study; C: cohort study; CoI: conflict of interest; CS: cross-sectional study; DA: dopamine agonists; DSM-IV: Diagnostic and Statistical Manual of Mental Disorders, Fourth Edition; F: female; FCI: Food Cravings Inventory; FU: follow-up; M: male; MIDI: Minnesota Impulse Disorder Interview; n: number; na: not available; ns: not specified; ORTO-15: Orthorexia Nervosa scale; QUIP: Questionnaire for Impulsive-Compulsive Disorders in Parkinson’s disease form; RCT: randomized clinical trial SD: standard deviation; USA: United States of America; y: years.

## Data Availability

Not applicable.

## Supplementary Materials

The following supporting information can be downloaded at: https://www.mdpi.com/article/10.3390/ijerph191610299/s1, Table S1. Search strategy adopted in each database, Table S2. List of excluded studies and reasons for exclusion, Table S3. The quality assessment of the included studies, in alphabetical order and based on study design.
