# Supplementary material for: Altered Food Behavior and Cancer: A Systematic Review of the Literature
Source: Int J Environ Res Public Health. 2022 Aug 18;19(16):10299. doi: 10.3390/ijerph191610299 (PMC9407804; doi:10.3390/ijerph191610299)
Supplement: Supplementary file 1 [file ijerph-19-10299-s001.zip › ijerph-1795795-supplementary.pdf]

Table S1. Search strategy adopted in each database

Database Searched	Date of Search	Search Strategy	N of Identified Records
PubMed/MEDLINE	19th April 2022	("Food Addiction"[MeSH Terms] OR "food craving"[Title/Abstract] OR "Compulsive Eating"[Title/Abstract] OR "Food Addictions"[Title/Abstract] OR "Orthorexia Nervosa"[MeSH Terms] OR "Orthorexia"[Title/Abstract]) AND ("neoplasms"[MeSH Terms] OR "Cancer Survivors"[MeSH Terms] OR "cancer"[Title/Abstract])	12
Scopus	19th April 2022	(TITLE-ABS-KEY ("neoplasms" OR "Cancer Survivors" OR cancer OR cancers OR neoplasm OR "cancer survivor")) AND (TITLE-ABS-KEY ("food addiction" OR "food craving" OR "compulsive eating" OR orthorexia OR "food addictions"))	60

Table S2. List of excluded studies and reasons for exclusion.

References	Reasons of Exclusion
Esquenazi et al. [35]	Authors investigate food addiction in patients with hamartoma, a non-neoplastic lesion
Johnson and Stephens [36]	Anaemia in pulmonary cancer survivor but due to a different cause
Speck et al.[37]	Taste alteration in cancer patients
Wildemberg et al. [38]	Review on prolactinomas that does not provide useful data on altered food behaviour

Table S3. The quality assessment of the included studies, in alphabetical order and based on study design

**Cross-Sectional/ Cohort Studies, Quality Assessed by Newcastle-Ottawa Scale**										
**Author Year [Ref]**	**Selection**				**Comparability**		**Outcome/Exposure^**			**Tot**
	Item 1	Item 2	Item 3	Item 4	Item 5a	Item 5b	Item 6	Item 7	Item 8	
Aslan et al. [12]	*	*	*	*	*	*	*	*	*	9/10
Dogansen et al. [41]	*	*	-	*	*	*	**	*	n.a.	9/10
Martinkova et al. [14]	*	-	-	*	*	-	*	-	n.a.	4/10
Shams-White et al. [13]	*	*	*	*	*	*	**	*	*	10/10
Vance, Campbell et al. [42]	*	*	-	*	-	-	*	-	n.a.	4/10

Bobonis Babilonia et al. [39]	JBI checklist for case report			
	Yes	No	Unclear	Not applicable
1. Were patient's demographic characteristics clearly described?	x	□	□	□
2. Was the patient's history clearly described and presented as a timeline?	x	□	□	□
3. Was the current clinical condition of the patient on presentation clearly described?	x	□	□	□
4. Were diagnostic tests or assessment methods and the results clearly described?	x	□	□	□
5. Was the intervention(s) or treatment procedure(s) clearly described?	x	□	□	□
6. Was the post-intervention clinical condition clearly described?	x	□	□	□
7. Were adverse events (harms) or unanticipated events identified and described?	x	□	□	□
8. Does the case report provide takeaway lessons?	x	□	□	□

Cohen et al. [40]	Risk of Bias-2 (RoB-2)
Randomized process	+
Deviation from intended interventions	+
Missing outocome data	+
Measurement of the outcome	+
Selection of the reported result	+
Overall bias	+

Low risk (+), some concerns (?), high risk (-)
